# Chiral topological excitons in the monolayer transition metal dichalcogenides

**DOI:** 10.1038/srep42390

**Published:** 2017-02-10

**Authors:** Z. R. Gong, W. Z. Luo, Z. F. Jiang, H. C. Fu

**Affiliations:** 1 College of Physics and Energy, Shenzhen University, Shenzhen, 518060, P. R. China

## Abstract

We theoretically investigate the chiral topological excitons emerging in the monolayer transition metal dichalcogenides, where a bulk energy gap of valley excitons is opened up by a position dependent external magnetic field. We find two emerging chiral topological nontrivial excitons states, which exactly connects to the bulk topological properties, i.e., Chern number = 2. The dependence of the spectrum of the chiral topological excitons on the width of the magnetic field domain wall as well as the magnetic filed strength is numerically revealed. The chiral topological valley excitons are not only important to the excitonic transport due to prevention of the backscattering, but also give rise to the quantum coherent control in the optoelectronic applications.

The topological states on the boundary or surface is one of the most fascinating phenomena in solid state physics. The electronic topological states have been extensively investigated both theoretically^1^,^2^,^3^,^4^,^5^ and experimentally^6^,^7^,^8^,^9^,^10^ based on the realization of the spin-orbit interaction in topological insulator. The topology protected conducting edge states have distinct properties from bulk states, and play important role in the electronic transport and facilitate the implementation of the topological states based electronic devices. The photonic topological states are also found in various systems such as microwave range photonic crystals^11^,^12^, the arrays of coupled optical resonators and waveguides^13^,^14^,^15^,^16^,^17^,^18^,^19^ and metamaterials^20^,^21^,^22^. It is motivated by the same idea of the electronic counterpart that the chiral edge states are prevent from backscattering and thus insensitive to disorder. Recently this idea has been generalized to the system consisting composite particles such as excitons and polarons^23^,^24^,^25^.

The topological states on the boundary of the 2D materials have attracted a lot of interests due to its 1D nature of the boundary^26^,^27^. It is the band inversion at the Dirac points that leads to the band structure of the topological states in 2D materials, such as graphene^28^,^29^,^30^,^31^,^32^,^33^. As a new member of the 2D materials family, the monolayer TMDs, realized in laboratories recently, shed light on the valleytronics, exciton physics and photoelectronic applications. The monolayer TMDs are direct bandgap semiconductors, where the conduction and valence band edges locate at the doubly degenerate corners of the Brillouin zone, as known as the Dirac points^34^,^35^,^36^. In fact there are two obstacles to generate electronic topological states in monolayer TMDs through the same mechanism in graphene: one is that as the direct gap semiconductor the TMDs possesses huge band gap (~1.6–2 eV); and another is that both the valence and conduction bands consist of the transition metal *d* orbitals on the same site, which prevents the staggered sublattice potential induced band inversion in graphene^32^. Those properties basically forbid the electronic band inversion in pristine TMDs.

Nevertheless, we can study the realization of the composite particles such as valley excitons instead of the electrons in the TMDs. For the bright excitons, there are two valley pseudospin configurations where the electron and hole both locate at either the K or −K valley^34^,^35^,^36^. The valley excitons follow optical selection rule, which means that the valley exciton locating at K(−K) valley only couples to *σ*^+^(*σ*^−^) circularly polarized light field^37^,^38^,^39^,^40^,^41^,^42^,^43^. Additionally, the Coulomb interaction between the electron and hole is exceptionally strong because of the 2D quantum confinement. The TMDs possesses valley degree of freedom, the valley-related optical selection rule and the strong Coulomb interaction, offering a new 2D system to explore the exciton physics. In addition, the two kinds of the valley excitons have opposite responses to the external out-of-plane magnetic field^44^, which implies a possible band inversion for valley excitons. In this sense, the TMDs provide a unprecedent platform to investigate the topological states of valley excitons.

In this paper, we shall theoretically investigate the chiral topological excitons emerging in the monolayer TMDs, where a bulk energy gap of valley excitons is opened up by a position dependent external magnetic field. The band dispersion of the bulk valley exciton realizes the massive Dirac cone, which exploits the strong valley-orbital coupling induced by Coulomb exchange interaction and the valley Zeeman splitting in the external magnetic field. For this unique Dirac cone of valley excitons, there are chiral topological exciton states emerging in the gap, whose number is determined by the bulk topological properties, i.e., Chern number = 2. Since the time reversal symmetry is broken by the magnetic field, there are only two different chiral topological exciton modes. We numerically reveal the dependences of the spectrum of the chiral topological excitons on the width of the magnetic field domain wall as well as the magnetic filed strength. The chiral topological valley excitons are not only important to the excitonic transport due to prevention of the backscattering, but also have potential application to the quantum coherent control in the optoelectronics.

## Results

### The bulk topological properties

The precise state of the valley exciton at K point is the superposition of the electron-hole pairs with all possible wave vector of the relative motion **q** and the definite wave vector of the center-of-mass motion **k**


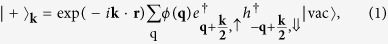


with the profile of relative motion *ϕ*(**q**), the electron (hole) creation operators *e*^†^(*h*^†^) and the electron (hole) spin up ↑ (spin down ⇓). The quantum state 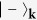 of the another valley exciton at −K point is the time reversal of the above one. In the low excitation limit of the valley excitons, the Hilbert space is spanned by the pseudospins described by the spin up(down) state 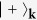 (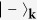). We start from the Hamiltonian of the valley excitons in the out-of-plane magnetic field


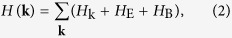


where 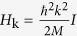 is the kinetic energy of the valley excitons, 
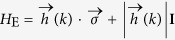
 is the valley-orbit coupling term with 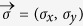, and *H*_B_ = −*g*_B_*μ*_B_*B*(**r**)σ_*z*_ is the position dependent valley Zeeman splitting. Here, *M* is total effective mass of the electron and hole consisting valley excitons, **k** = (*k*_*x*_, *k*_*y*_) = (*k*cos*θ, k*sin*θ*) and **r** = (*x, y*) are the wave vector and the position coordinate of the valley excitons’ center-of-mass motion respectively, *μ*_B_ is the Bohr magneton, *I* and σ_*α*_(*α* = *x, y, z*) are respectively the identity matrix and Pauli matrices, and *B*(**r**) is the position dependent magnetic field.

The valley-orbit coupling actually introduces the inter-valley transition, where the effective field 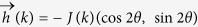 originates from the long range part of the Coulomb exchange interaction^45^, where *J(k*) = *Jk/K* is the valley orbit coupling strength scaling linearly of the wave numbers *k*, and *K* = 4*π*/3*a* is the wave vector from the K to Γ point of the Brillouin zone. The constant 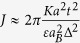 only depends on the parameters of the monolayer TMDs, the lattice constant *a*, the hooping constant *t*, the effective dielectric constant *ε*, the Bohr radius of the exciton *a*_*B*_ and the bang gap Δ.

The magnetic response of the valley excitons, caused by the out-of-plane magnetic field, gives rise to the last term *H*_B_. Since the valley exciton is roughly regarded as a bounded electron-hole pair, both the electron and hole magnetic moments contribute to the exciton’s Zeeman splitting in the magnetic field, leading to the effective g-factor of the valley excitons


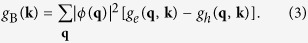


**Figure 1:**
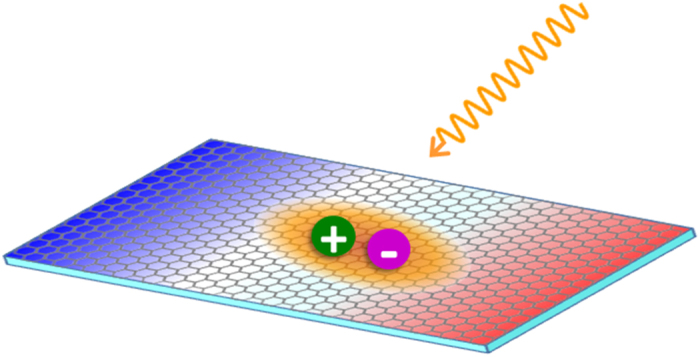
Carton of generating the topological exciton in the vicinity of the magnetic field domain wall. The orange wave line represents the pumping light field. The wavefuntion of its relative motion is represented by the orange area, in which the electron and the hole are respectively represented by the purple sphere with “−” symbol and the green sphere with “+” symbol. The colored region of the monolayer TMDs represent the position dependent magnetic field.

The minus sign before the hole’s g factor originates from the electron-hole duality in the semiconductor. Both the transition metal *d* orbital and valley magnetic moments contribute to the Lande g-factor of electron and hole. In the recent experiment based on WSe_2_, the typical measured *g*_*B*_ is about 1.8^44^. In this sense, the typical valley Zeeman splitting is about several meV when the applied magnetic field is up to 10*T*. Since the momentum scale of the profile *ϕ(**q***) is about the reciprocal of the of Bohr radius of the valley exciton, which is much smaller than the momentum scale of the Lande g-factor of electron and hole. In this sense, we obtain the approximate effective Lande g-factor for the valley excitons *g*_B_ ≈ *g*_B_(**k** = 0), which becomes independent of the wave vector of the center-of-mass motion. The *σ*_*z*_ term in the *H*_B_ implies that the valley Zeeman energies are exactly opposite at K and −K points, which results from the valley-index-dependent magnetic moments of the valley excitons. The setup is schematically shown in Fig. 1. It is noticed that the inhomogeneity of the magnetic field *U(r*_*α*_)(*α* = *e, h*) shifts the momentum of the electron and hole and eventually cause the overlap of their wavefunctions, which gives rise to an additional inter-valley coupling of single states. It is straightforwardly calculated as


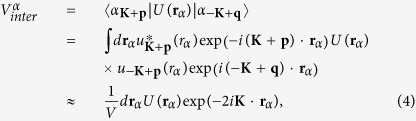


where 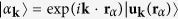 are the Bloch wave functions of the electron and hole. In the last step we apply the condition 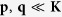. For a slowly varying magnetic field domain wall with a typical length of domain wall more than hundreds of lattice constant, the inter-valley couplings of the single states are about 10^−5^*E*_*B*_, where *E*_*B*_ is the Zeeman energy. Obviously, they are sufficiently small. Additionally, since the inter-valley coupling of the excitons basically are the summation of the inter-valley coupling of single states, both of them are neglected in the following discussion.

**Figure 2:**
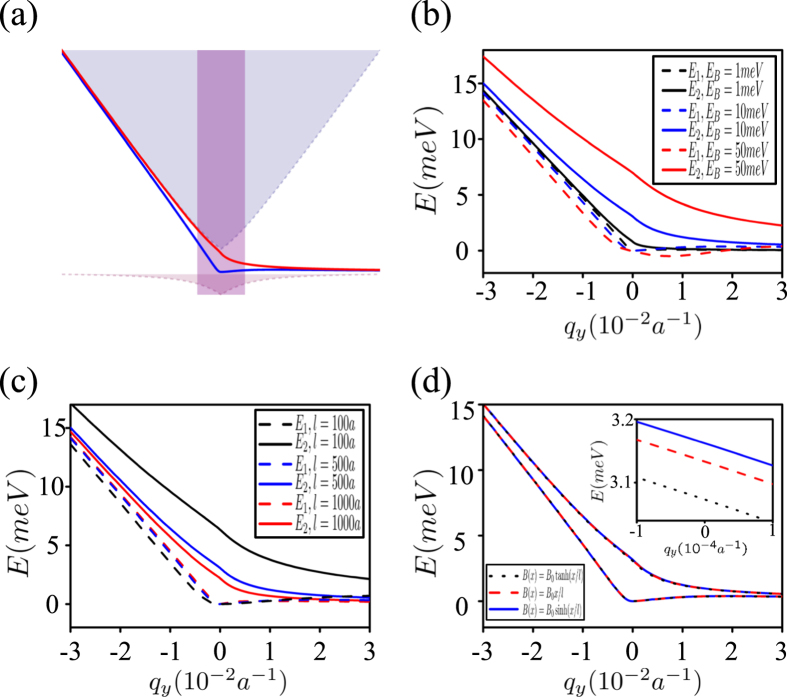
(**a**) Typical spectrum of the topological excitons, which are denoted by the red and blue solid lines. Here, the light blue, light red and purple region respectively denotes the upper band, the lower band and the light cone of the pumping light field. (**b**) The spectrum of the topological excitons versus *q*_*y*_ for different magnetic field strength. (**c**) The spectrum of the topological valley excitons versus *q*_*y*_ for different widths of the magnetic domain wall. (**d**) The spectrum of the topological excitons versus *q*_*y*_ for different types of the magnetic domain wall. Insert: magnified view of the spectrum. See text for the details.

In the bulk, the Hamiltonian can be written as a matrix form^46^


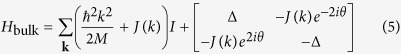


on the basis 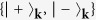, where Δ = *g*_B_*μ*_B_*B* is the valley Zeeman energy. The valley Zeeman energy approximately takes the fixed value because we only consider the bulk topological properties at the region where the applied magnetic field is homogeneous. The valley exciton dispersion splits into two branches with energies


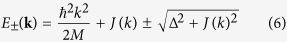


and corresponding eigen-wavefunctions


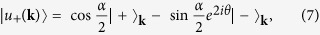



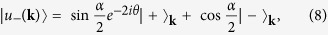


where 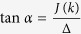. So it realizes a massive Dirac cone. The valley Zeeman energy plays the role of the mass in the Dirac-like equation and opens up a gap between two bands of valley excitons (see Fig. 2(a)). As composite particles, the valley excitons still share the same Brillouin zone of the electron and hole in the monolayer TMDs. In contrast of the Dirac cones of the electron which locates at the corner of the Brillouin zone, the unique Dirac cone of the valley exciton locates at the center of the Brillouin zone.

In order to describe the bulk topological property of the valley excitons, the Berry connection 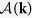 and Berry curvature Ω(**k**), defined respectively as


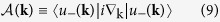



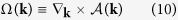


are introduced as the gauge potential and the gauge field of the lower valley exciton band. The Berry curvature is regarded as a magnetic field in the valley exciton center-of-mass momentum space, the integral of which over the **k**-space area gives rise to the Berry phase of the valley exciton if it adiabatically go around the area boundary. The Chern invariant is defined as the flux of the Berry curvature threading the entire Brillouin zone


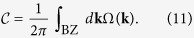


For the valley excitons described by equation (2), one find the Berry curvature centered at the Dirac cone


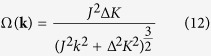


and thus the Chern invariant 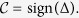 The nonzero Chern number implies the existence of topology states of valley excitons.

When the position dependent magnetic field is applied, it leads to the band inversion of the valley excitons, and the number of the topological charge equals to the difference of the bulk topological charges on the both side of the domain wall^32^, which reads 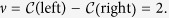 Therefore, it is imaginable that two topological states will emerge at the vicinity of the magnetic field domain wall. Since the magnetic field breaks the time reversal symmetry and the Dirac cone is uniquely centered at zero momentum point, such topological states become chiral ones without time reversal symmetry.

For the sake of simplicity, we assume the position-dependent magnetic field varies only along *x*-direction, namely *B*(**r**) ≡ *B(x*). When the magnetic field varies slowly along *x*-direction, the wavefunction of the topological exciton can be written as a two-component vector


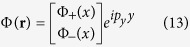


where Φ_±_(*x*) are the wavefunction profile, and *p*_*y*_ is the *y*-component momentum. The wavefunction profile Ψ(*x*) = {Φ_+_(*x*), Φ_−_(*x*)}^T^ satisfies the following equation


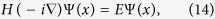


where the momentum in the original Hamiltonian is substituted by the operator in the real space as **k** → −*i*∇. According to the symmetry analysis of the above equation, there are two solutions 
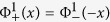
 and 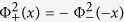 corresponding to two topological excitons. However, the chirality index of the valley exciton Dirac cone equals to 2 in contrast with the well known Dirac cone, and the off-diagonal element ∝ *ke*^−2*iθ*^ does not possess a simple operator form in the real space. So it is appropriate to solve the equations in the momentum space numerically. The details is presented in the Method section.

### Numerical results

In order to confirm the existence of two chiral topological excitons, we numerically evaluate the energy spectrum and the corresponding wavefunctions from equation (14). Here the constant *J* is chosen as 1 eV, and the lattice constant for MoS_2_ is 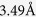. In Fig. 2(a–c), the magnetic field domain wall is assumed as 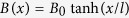. The typical spectrum of the topological excitons are demonstrated in Fig. 2(a). Obviously there are two different topological excitons, which are consistent with the Chern number. When *q*_*y*_ tends to positive (negative) infinity, the dispersion of both topological excitons is convergent to the edge of the conduction (valence) band. The dependence of the spectrum on the magnetic field strengthes, widths and types of the magnetic domain wall is depicted in Fig. 2(b–d). In Fig. 2(b) and (c), the solid and dashed lines corresponds to the first and the second solution of the equation of wavefunction profile. The red, blue and black lines in Fig. 2(b) correspond to different magnetic valley Zeeman energy *E*_*B*_ ≡ *g*_B_*μ*_B_*B*_m*ax*_ = 1 meV, 10 meV, 50 meV and *l* = 500*a* with *a* the lattice constant. Figure 2(c) corresponds to different width of the magnetic domain wall *l* = 100*a*, 500*a*, 1000*a* and *E*_*B*_ = 10 meV, respectively. Obviously, the smaller the width of magnetic domain wall is, or the stronger the magnetic field strength is, the larger the energy difference between two topological excitons becomes. It actually results from the stronger quantum confinement of the magnetic domain wall, which suggests to adjust the spectrum of the topological excitons through both the magnetic field strength and the width of the magnetic domain wall. We compare the different types of the magnetic domain wall and find their spectrum almost coincide, which results from the similar behavior of those functions in the vicinity of *x* = 0 as 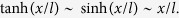 It implies that the topological excitons basically locates at the vicinity of the magnetic domain wall.

**Figure 3:**
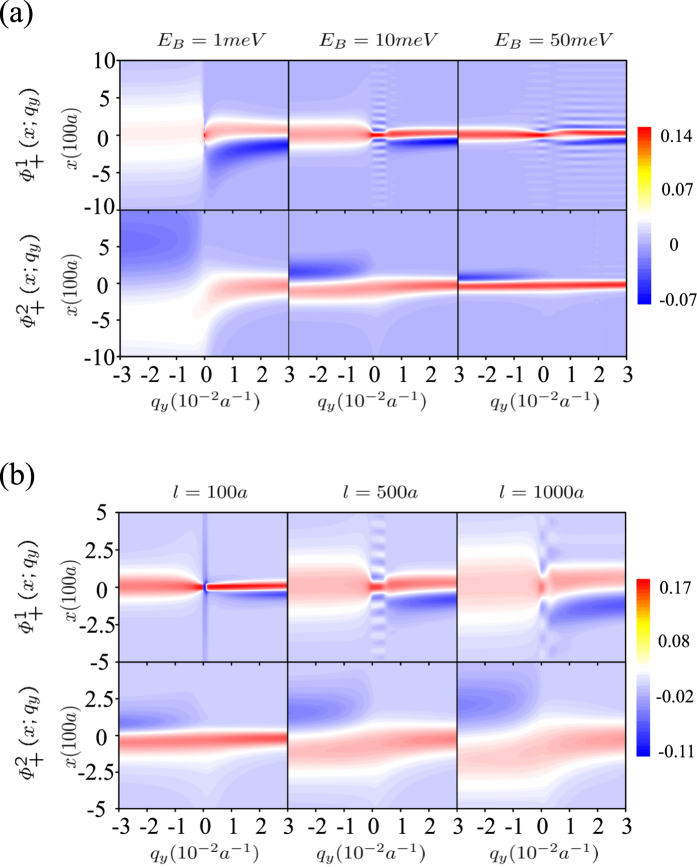
Contour plot of the wavefunction profile 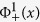 and 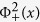 versus *x* and *q*_*y*_ for (**a**) different widths of the magnetic field domain wall and (**b**) different magnetic field strengths. Obviously under stronger quantum confinement with smaller widths of the magnetic field domain wall or stronger magnetic field strengths, the topological valley excitons becomes more local in the vicinity of the domain wall.

We also present the corresponding wavefunctions in Fig. 3. Since the four components of the two solutions are related to each other as 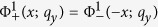 and 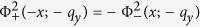, we only present the contour plot of wavefunction profile 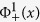 and 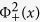 versus *x* and *q*_*y*_ in Fig. 3. Parameters in Fig. 3(a) and (b) are the same as ones in Fig. 2(b) and (c). Obviously under stronger quantum confinement with smaller width of the magnetic field domain wall or stronger magnetic field strength, the topological valley excitons become more local in the vicinity of the domain wall.

## Discussion

The optoelectronic properties of the topological excitons depend on their optical dipole defined as 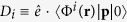, *i* = 1, 2, where 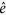 is the polarization of pumping light field and **p** is the electric dipole moment. Since the relative and center-of-mass motions are independent for the topological exciton, the optical dipole can be factorized as *D*_*i*_ = *A*_*i*_(*D*_+_ + (−1)^*i*+1^*D*_−_), where 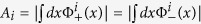 are the integrals of the wavefunction profile and 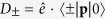 are the optical dipole for the valley excitons 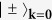. It indicates that the first and second topological excitons exactly inherit the optical selection rules from the linear combinations of the valley excitons 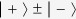, which means that the two topological excitons can be initialized by utilizing the linear-polarized pumping light fields along *x*- and *y*-direction, respectively. Additionally, the optical dipole are adjustable by tuning the magnetic field because *A*_*i*_ depends on the widths of the magnetic field domain wall and magnetic field strength. These two topological excitons possess controllable gap (∼ meV), optical initialization and robust transportation protected by topology, and thus may shed light on the quantum coherent optoelectronic devices based on these topological excitons in TMDs.

The typical time to generate the valley excitons is much shorter than the the lifetime of the valley excitons, which is about tens of ps for monolayer TMDs. Additionally, the optical dipole of the topological chiral excitons can be adjusted by the applied magnetic field, which suggests a longer lifetime for the topological excitons. Therefore, the lifetime of the excitons would not affect the generation of the topological excitons. The longer lifetime allows more subtle control, which may facilitates the application of the optoelectronics based on the topological excitons.

## Methods

The spectrum and the corresponding wave-functions of the chiral topological excitons can be obtained by solving equation (14). Actually, the quadratic term together with the linear dispersion from the massless Dirac equation gives rise to energy minimum around 
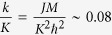
 corresponding to the minimum energy 
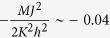
eV, which is much larger than the magnetic field induced Zeeman splitting. Therefore with the position dependent magnetic field *B*(**r**) and considering the emergent edge state around Dirac point, we drop the quadratic terms and obtain the Hamiltonian of topological excitons as


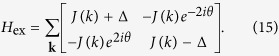


Additionally, we define parameters *K*_*B*_(*x*) = *Kg*_*E*_*μ*_*B*_*B(x*)/*J* and *K*_*E*_ = *EK/J*. The Schrödinger equation for the wave-function profile Φ_±_(*x*) becomes


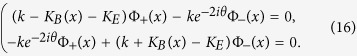


Although the momentum operator can be written as *ke*^*iθ*^ = *k*_*x*_ + *ik*_*y*_ = −*i*∂_*x*_ + ∂_*y*_ in the real space, the operators *k* and *ke*^−2*iθ*^ do not possess a simple operator form. However, they are classical numbers in the momentum space. Thus it is convenient to solve above eigen-equations in the momentum space. By applying the Fourier transformation, the equation (16) becomes


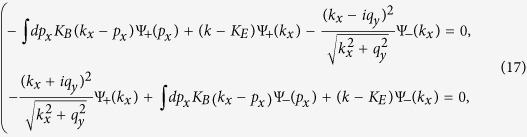


The convolution terms means all the equations for different *k*_*x*_ are coupled to each other. If we discretize the momentum *k*_*x*_, all the equations are linear and thus the eigenvalues *K*_*E*_ and the corresponding wavefunctions can be obtained numerically.

## Additional Information

**How to cite this article:** Gong, Z. R. *et al*. Chiral topological excitons in the monolayer transition metal dichalcogenides. *Sci. Rep.* **7**, 42390; doi: 10.1038/srep42390 (2017).

**Publisher's note:** Springer Nature remains neutral with regard to jurisdictional claims in published maps and institutional affiliations.
